# A Rare Cause of Recurrent Encephalopathy in a Septuagenarian: A Case Report

**DOI:** 10.7759/cureus.41015

**Published:** 2023-06-27

**Authors:** Sambhaji Pawal, Rahul Arkar

**Affiliations:** 1 Department of Interventional Radiology, Dr. D. Y. Patil Medical College, Hospital & Research Centre, Pune, IND

**Keywords:** abernethy malformation, endovascular embolization, triphasic cect, ammonia, hepatic encephalopathy

## Abstract

The Abernethy malformation is an extremely rare congenital, extrahepatic, portosystemic shunt. There are many problems associated with this abnormal portovenous shunting and subsequent reduced hepatic portal venous flow. With the advances in non-invasive imaging technologies, these cases are diagnosed in more numbers; however, the presentation of patients is varied and the natural history is not completely known. The presenting symptom of the portosystemic shunt is mainly hyperammonemia, leading to encephalopathy. Management varies depending on the type of shunt and its clinical course; hence, the classification of the congenital portosystemic shunt is important in these patients.

## Introduction

When there is an abnormal communication between the portal and systemic venous circulation, it is called an abnormal portosystemic shunt. Normally, blood from the mesenteric region is drained to the liver for processing and detoxification. When an abnormal portosystemic shunt is present, the blood from mesenteric circulation bypasses the liver and reaches without detoxification into the systemic circulation. As such, these abnormal portosystemic shunts can be either congenital or may be acquired in nature. Further, these congenital shunts can be either extrahepatic or intrahepatic. Abernethy described a congenital extrahepatic portosystemic shunt (CEPS) [1], hence, the name Abernethy malformation which is a rare occurrence in adulthood and old age.

## Case presentation

We report a case of recurrent encephalopathy in a 72-year-old female patient. She was admitted to our hospital for mild jaundice with associated comorbidities of diabetes mellitus and hypothyroidism. There was no history of alcoholism. Her liver function tests were relatively normal with the exception of a bilirubin level of 1.85 mg/dL. Her blood ammonia level was elevated. The hemogram was normal. The coagulation profile was normal. Viral markers such as hepatitis B surface antigen and hepatitis C virus were negative.

**Figure 1:**
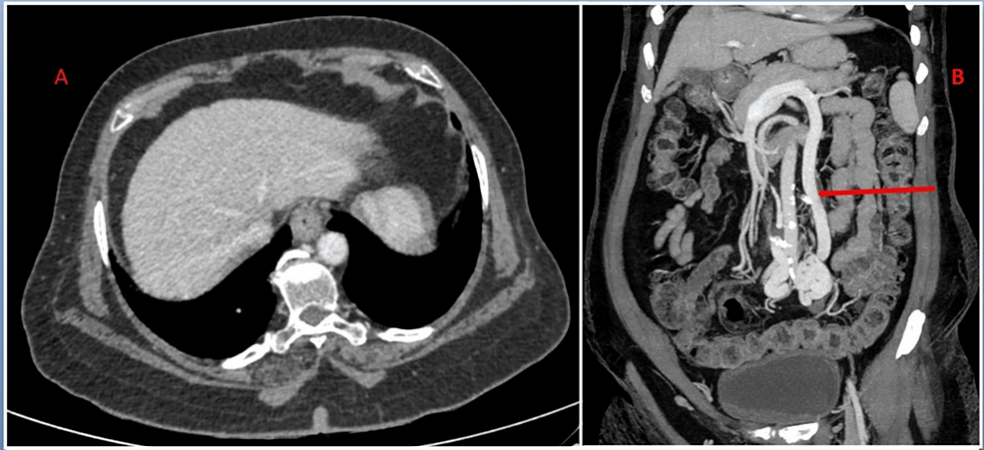
(A) Axial CT showing mild surface nodularity of the liver. (B) Coronal MIP CT image in the venous phase shows abnormal venous shunt (red arrow) connecting the extrahepatic portal vein to the left common iliac vein. CT: computed tomography; MIP: maximum intensity projection

**Figure 2:**
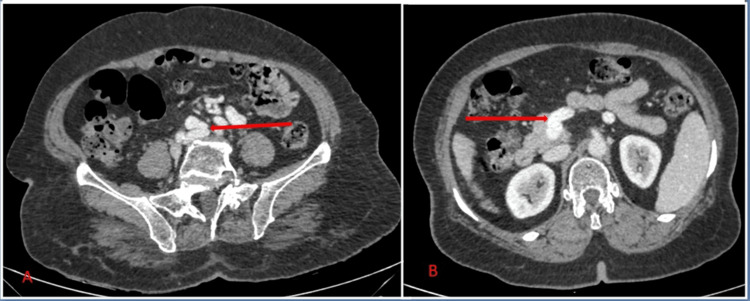
(A) Distal end (red arrow) of the abnormal venous shunt terminating in the left common iliac vein. (B) Proximal end (red arrow) of the abnormal venous shunt originating from the extrahepatic portal vein.

Tri-phasic contrast-enhanced computed tomography (CECT) of the abdomen and pelvis was performed. CECT revealed abnormal venous communication between the extrahepatic portal vein and the systemic circulation into the left common iliac vein. This abnormal vein showed extensive tortuosity near the iliac vein end (Figures 1, 2). The superior mesenteric vein and splenic vein appeared normal. There was a relatively small caliber of intrahepatic portal vein.

Venous phases of CECT were analyzed in multiple reformats which showed the abnormal long dilated shunt vessel after the confluence of the superior mesenteric vein and splenic vein and the left common iliac vein. The liver showed mild surface nodularity (Figure 1). The spleen was normal in size. There were no ascites.

The patient was admitted to the medical gastroenterology division and managed conservatively with a low-protein diet. She was also given adequate hydration, enema, and lactulose. The serum ammonia level improved, and the patient was discharged in stable condition.

## Discussion

Diagnosis

Ohwada et al. [2] proposed the criteria for the diagnosis of the congenital portosystemic shunt. The criteria include the following parameters: absence of hypersplenism or portal hypertension, absence of microscopic changes of cirrhosis/hepatitis/portal hypertension, small diameter of the intrahepatic portal vein with no evidence of arteriovenous fistula, and/or no history of abdominal surgery or inflammation.

The primary diagnosis of CEPS is challenging in old patients as the existence of chronic liver disease is more common. Encephalopathy caused by chronic hyperammonemia is a common presentation. Another important aspect of CEPS is that features such as gastroesophageal varices, ascites, or enlarged spleen which represent portal venous hypertension are generally not seen [3,4]. Hence, special attention should be paid to note encephalopathy, especially in older patients. Our case represents the old-age-onset encephalopathy associated with the congenital portosystemic shunt.

Classification

Broadly, Abernethy malformations [5] are of two types, namely, type I in which end-to-side shunt is seen, and type II in which side-to-side shunt is seen.

Type I shows the absence of intrahepatic portal divisions and is subdivided into Ia and Ib. Type Ia represents separate drainage of the superior mesenteric vein and splenic vein into the systemic vein. Type Ib represents a common trunk formed by the superior mesenteric vein and splenic vein before an abnormal drainage pattern.

Type II shows hypoplastic intrahepatic portal vein with some part of portal venous blood drained into the systemic vein via side-to-side extrahepatic communication.

The World Congress of Gastroenterology at its 11th meeting formed a consensus group for standardized nomenclature for hepatic encephalopathy. According to this group, hepatic encephalopathy type B refers to encephalopathy in the presence of a portosystemic shunt and no hepatocellular abnormality [6].

Portosystemic encephalopathy is characterized by high levels of ammonia in the blood contributed by the digestive system, which is normally metabolized in the liver. The excess ammonia in systemic circulation affects the brain and is toxic to astrocytes as well as neurons leading to symptoms of encephalopathy [7].

The percentage of encephalopathy associated with congenital portosystemic shunts differs according to different studies. Kobayashi et al. [8] found 13.2% of patients with congenital portosystemic shunts to have portosystemic encephalopathy.

CEPS may go unnoticed in many patients unless associated with hepatic or cardiac abnormalities, as reported by Morgan et al. [5].

Generally, the presentation of these CEPS shunts is in middle or old age. However, this was contradicted by Murray et al. [9] who documented a younger median age for type II CEPS which was two years. For more detailed and elaborative discussion and evaluation, studies with large sample sizes are needed.

Opinions differ on the presentation and underlying pathology. The initial presentation of a patient can be encephalopathy where the exact etiology is not known. During the course of evaluation of encephalopathy of unknown origin, the investigation might show vascular anomalies such as CEPS, as in the case of our patient. However, it is challenging to establish the diagnosis in such a case because both clinicians and radiologists need to be sensitized about this rare entity. Furthermore, clinicians need to be aware of such an entity with subtle presentations. Of note, the hallmark is to identify abnormally elevated blood ammonia levels. The aging brain cannot tolerate elevated blood ammonia levels, and in such cases, the presentation of symptoms is more likely with old age [10].

The important factors responsible for the type and nature of symptoms associated with portosystemic encephalopathy of CEPS origin are the age of the patient and the shunt ratio. As reported by Uchino et al. [11], a shunt ratio of more than 60% predisposed patients to encephalopathy in patients with CEPS. The isotope iodine 123-iodoamphetamine administered rectally into the sigmoid colon is promptly absorbed into the inferior mesenteric vein and is carried to the liver. When a portosystemic shunt is present, the isotope can be detected in both the liver and lungs.

The shunt ratio is calculated by dividing counts obtained in the lung by the total counts obtained in the liver and lungs (normal: <5%). The patients are classified into three groups depending on the percentage of shunt. Group A comprises patients with a shunt ratio of less than 30%, group B comprises patients with a shunt ratio of 31-60%, and group C patients have a shunt ratio of more than 60%. Generally, patients in group A have no symptoms throughout life. Patients in group C present at a very early age in life, as observed in a three-year-old child with a patent ductus venosus. The oldest patient who did not have hepatic encephalopathy was a 72-year-old woman with an intrahepatic portosystemic venous shunt with a shunt ratio of 30% [11].

There are various classification systems for congenital portosystemic venous shunts. The anatomical classification proposed by Morgan and Superina [5] is the most commonly used. It divides the shunt into intrahepatic and extrahepatic (CEPS) types with additional components depending on the presence or absence of the intrahepatic portal vein. The CEPS type is characterized by the connection between a systemic vein and porto-mesenteric vasculature before the division of the portal vein at the hilum of the liver. The spectrum of the draining systemic vein is variable such as the inferior vena cava (most common) [9], renal, iliac, azygous vein, or even right atrium.

In the study reported by Murray et al. [9], there was significant female predominance (74%) in type I CEPS with no significant female preponderance in type II. However, the study mentioned a bias of misclassification of some type II cases of CEPS being classified as type I. Hence, there is definitely a question regarding the true gender incidence [9].

As in our case, when the presentation of patients is in old age, the challenges are from the perspective of diagnosis and management with long-term hyperammonemia and recurrent encephalopathy. The scenario becomes more difficult when confounding factors such as alcoholism are present. In such a scenario, the diagnosis of CEPS is not considered in the differential diagnosis by clinicians and even radiologists. Considering the encephalopathy, all investigations are done pertaining to the brain and CEPS can go undetected if abdominal imaging is not requested. Our case is a similar example wherein after the tri-phasic CECT scan, the diagnosis of type II shunt was established.

There are few associations with Abernethy malformations, for example, focal nodular hyperplasia and malignancy such as hepatocellular carcinoma.

Embryology

Systemic veins arise from the anterior cardinal veins and posterior cardinal veins. Extra-embryonic vitelline veins with a complex interplay with umbilical veins give rise to the portal venous system. Aberrations in the development of the portal venous system give rise to complex anomalies. The development of CEPS is explained by the complex development of the vena cava, vitelline veins, and any abnormality in their development [12].

Treatment

The treatment strategy in cases of congenital portosystemic shunts depends on the type of shunt, age of the patient, progression of the disease, and presence of any other comorbidity. Hence, the classification of congenital portosystemic shunts is the key to guiding and determining the correct course of managing the patient. The absolute indications of treatment are hepatopulmonary shunt and portopulmonary hypertension [13]. Traditionally, liver transplantation is considered for type I CEPS patients. Endovascular embolization is preferred for patients with type II CEPS [14].

Limitations

In our case, the patient was doing well with medical management. Hence, after a discussion with the medical gastroenterology team and the patient’s family, it was decided to continue medical management. Therefore, endovascular embolization was not performed in our case at this stage. We consider this a limitation of our case report.

## Conclusions

Abernethy malformation is a rare cause of hepatic encephalopathy in older patients. Both clinicians and radiologists should be sensitized and aware of congenital extrahepatic portosystemic shunts as an etiology of hyperammonemia and associated hepatic encephalopathy. Meticulous tri-phasic CECT scan improves diagnostic accuracy and helps in classifying patients in view of management.

## References

[1] Abernethy LJ (2003). Classification and imaging of vascular malformations in children. Eur Radiol.

[2] Ohwada S, Hamada Y, Morishita Y (1994). Hepatic encephalopathy due to congenital splenorenal shunts: report of a case. Surg Today.

[3] Webb LJ, Sherlock S (1979). The aetiology, presentation and natural history of extra-hepatic portal venous obstruction. Q J Med.

[4] Alonso-Gamarra E, Parrón M, Pérez A, Prieto C, Hierro L, López-Santamaría M (2011). Clinical and radiologic manifestations of congenital extrahepatic portosystemic shunts: a comprehensive review. Radiographics.

[5] Morgan G, Superina R (1994). Congenital absence of the portal vein: two cases and a proposed classification system for portasystemic vascular anomalies. J Pediatr Surg.

[6] Dharel N, Bajaj JS (2015). Definition and nomenclature of hepatic encephalopathy. J Clin Exp Hepatol.

[7] Bleibel W, Al-Osaimi AM (2012). Hepatic encephalopathy. Saudi J Gastroenterol.

[8] Kobayashi N, Niwa T, Kirikoshi H, Fujita K, Yoneda M, Saito S, Nakajima A (2010). Clinical classification of congenital extrahepatic portosystemic shunts. Hepatol Res.

[9] Murray CP, Yoo SJ, Babyn PS (2003). Congenital extrahepatic portosystemic shunts. Pediatr Radiol.

[10] Kerlan RK Jr, Sollenberger RD, Palubinskas AJ, Raskin NH, Callen PW, Ehrenfeld WK (1982). Portal-systemic encephalopathy due to a congenital portocaval shunt. AJR Am J Roentgenol.

[11] Uchino T, Matsuda I, Endo F (1999). The long-term prognosis of congenital portosystemic venous shunt. J Pediatr.

[12] Howard ER, Davenport M (1997). Congenital extrahepatic portocaval shunts--the Abernethy malformation. J Pediatr Surg.

[13] Franchi-Abella S, Branchereau S, Lambert V (2010). Complications of congenital portosystemic shunts in children: therapeutic options and outcomes. J Pediatr Gastroenterol Nutr.

[14] Woodle ES, Thistlethwaite JR, Emond JC, Whitington PF, Vogelbach P, Yousefzadeh DK, Broelsch CE (1990). Successful hepatic transplantation in congenital absence of recipient portal vein. Surgery.

